# An Insight into the Difficulties in the Discovery of Specific Biomarkers of Limbal Stem Cells

**DOI:** 10.3390/ijms19071982

**Published:** 2018-07-06

**Authors:** Zhi Hou Guo, Wei Zhang, Yang Yan Sheng Jia, Qing Xiu Liu, Zhao Fa Li, Jun Sheng Lin

**Affiliations:** School of Medicine, Huaqiao University, Quanzhou 362021, China; 1601116005@hqu.edu.cn (Z.H.G.); weizhang1992@hqu.edu.cn (W.Z.); 1234111009@hqu.edu.cn (Y.Y.S.J.); 17011071006@hqu.edu.cn (Q.X.L.); lizhaofa@hqu.edu.cn (Z.F.L.)

**Keywords:** limbal stem cell (LSC), LSC niche, LSC biomarker, heterogeneity

## Abstract

Keeping the integrity and transparency of the cornea is the most important issue to ensure normal vision. There are more than 10 million patients going blind due to the cornea diseases worldwide. One of the effective ways to cure corneal diseases is corneal transplantation. Currently, donations are the main source of corneas for transplantation, but immune rejection and a shortage of donor corneas are still serious problems. Graft rejection could cause transplanted cornea opacity to fail. Therefore, bioengineer-based corneas become a new source for corneal transplantation. Limbal stem cells (LSCs) are located at the basal layer in the epithelial palisades of Vogt, which serve a homeostatic function for the cornea epithelium and repair the damaged cornea. LSC-based transplantation is one of the hot topics currently. Clinical data showed that the ratio of LSCs to total candidate cells for a transplantation has a significant impact on the effectiveness of the transplantation. It indicates that it is very important to accurately identify the LSCs. To date, several putative biomarkers of LSCs have been widely reported, whereas their specificity is controversial. As reported, the identification of LSCs is based on the characteristics of stem cells, such as a nuclear-to-cytoplasm ratio (N/C) ≥ 0.7, label-retaining, and side population (SP) phenotype. Here, we review recently published data to provide an insight into the circumstances in the study of LSC biomarkers. The particularities of limbus anatomy and histochemistry, the limits of the current technology level for LSC isolation, the heterogeneity of LSCs and the influence of enzyme digestion are discussed. Practical approaches are proposed in order to overcome the difficulties in basic and applied research for LSC-specific biomarkers.

## 1. Introduction

The cornea is the outermost tissue of the eye which transmits light for vision [1]. Keeping the integrity and transparency of the cornea is very important for ensuring normal vision. Statistics data from the World Health Organization (WHO) showed that more than 400 million people are suffering from visual impairment worldwide, and about 40 million of the population are blind, of which 10 million are caused by corneal diseases or injury [2,3]. It seriously impacts the sufferer’s life quality and causes a heavy social and economic burden.

The dynamic stability of cornea epithelium, composed of multiple layers of squamous and stratified epithelium, is maintained by the corneal epithelium stem cells within limbus, the transmission area between the cornea and sclera [1,4,5,6]. The corneal limbal stem cells (LSCs) are located in the basal layer of limbus epithelium. The variety of LSCs quantities and bio-properties can directly affect the homeostasis of corneal epithelial cells (CECs) and thus affect the visual function [7,8]. There are many factors including chemical burns and incorrectly wearing contact lenses, and surgery can cause corneal damage, even leading to the limbal stem cell deficiency (LSCD) [9]. If the damaged cornea cannot be repaired in time due to lack of LSCs, it would cause inflammation, neovascularization, ulceration, conjunctivalization, corneal opacification, etc. [10,11]. In recent years, the rapid development of regenerative medicine has promoted the treatment of corneal disease, especially the specificity- and target-based corneal transplantation [12]. The inadequate resources of cornea, immune rejection, and lifelong administration of immunosuppressive drugs have limited the application of corneal transplantation, thereby LSC-based therapeutics have become a hot topic [13,14].

The transplantation of autografts, prepared either by the limbal epithelial cells expanded ex vivo using a xeno-free explant culture system or by limbal biopsy sampled from the unaffected eye from the patient, has been applied in the clinic [15,16]. The transplantation of allografts is still a challenge. The clinical data showed that the ratio of LSCs to the candidate cells for transplantation has a significant impact on the efficacy of LSC-based therapeutics [17]. It indicates that it is important to accurately identify the LSCs. LSCs account for a small population in limbal epithelial cells (LECs) [18], which limited its application in the treatment of LSCD. Although direct pluripotent stem cells (PSCs) and embryonic stem cells (ESCs) differentiated into LECs for the treatment of LSCD have been reported [19,20], the differences in immunogenicity between candidate donated ESCs and their recipients [21] and the purity of desired cells derived from PSCs are challenges [22]. Induced pluripotent stem cells (iPSCs), demonstrated from adult fibroblasts by introducing four factors (*oct3/4*, *sox2*, *c-Myc*, and *klf4*), was first proposed by Shinya Yamanaka in 2006 [23]. Unlike adult stem cells, iPSCs exhibit a self-renewing property for many passages, promising to obtain enough iPSCs for further clinical therapeutic purposes [21,24]. Recently, iPSC technology has been reported in cell-based therapy of LSCD treatment [24,25]. Due to their lesser restrictions, iPSC technology might be a new method to overcome the limitations of LSCs for a wider application in the treatment of LSCD. However, the problems of the well-known line-to-cell line heterogeneity and variability in the differentiation capacity of individual cell lines still remain, which significantly impact the success of the cell-based therapeutic strategy [26]. Therefore, it is still necessary to find the specific biomarkers of LSCs for enriching the high purity of desired cells derived from the iPSC population. The recognition of LSCs mainly depends on the presence or absence of specific biomarkers. To date, LSC putative biomarkers have been widely reported, but none of the biomarkers showed LSC-specificity, resulting in it being hard to establish a robust protocol to identify LSCs.

## 2. LSC and LSC Niche

**Figure 1:**
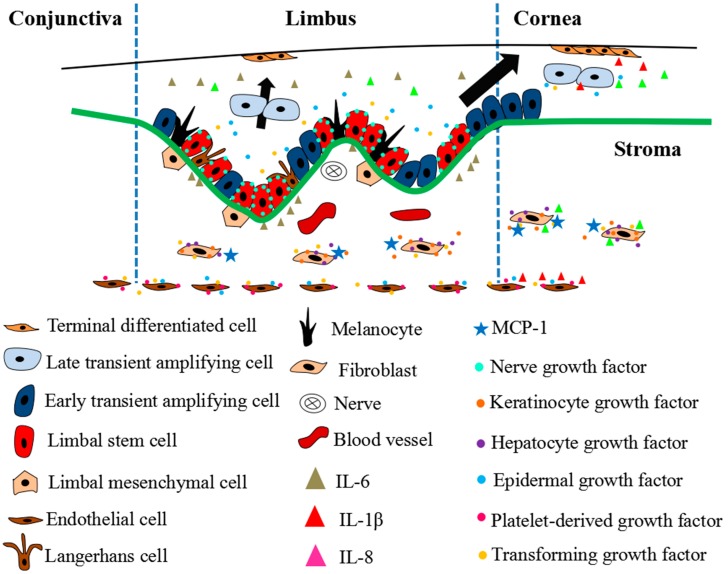
Schematic of the LSC niche. The LSC niche located at Vogt in limbal epithelium. There are several types of cells including limbal stem cells, early transient amplifying cells, melanocytes and langerhans cell within the niche. The limbal stroma, which is highly innervated and vascularized, is located underneath the basement membrane (BM). In limbal stroma, the mesenchymal cells can be found in the limbal stroma and play an important role in the LSC niche. Cyotokines play a role of mediator for cell–cell communication within the niche.

LSC, a subpopulation of limbal epithelial cells, has unique characteristics, such as small size, a large nucleus-to-cytoplasm (N/C) ratio, slow cell cycle, short-period of DNA synthesis, high-potential of differentiation, proliferation, etc. [27,28,29]. LSCs normally reside in the limbal palisades of Vogt. The niche of LSCs (Figure 1) consists of cells, extracellular matrix, and functional mediators, such as secreted cytokines, providing a microenvironment for LSCs to play their roles, such as self-renewal [30,31].

### 2.1. Cells

The cells involved in the LSC niche include not only LSCs, but also transient amplifying cells (TACs), melanocytes, langerhans cells, etc. [1]. Except for the pathological conditions, such as pterygium, the melanocytes are specifically located at limbal epithelium. They protect eyes from UV ray damage by generating pigments, and play roles in free radicals scavenging and intraocular pressure sensing [32,33,34]. Melanocytes are colocated with LSCs by the homophilic binding interaction of N-cadherin in an LSC niche, which plays an important role in the maintenance of LSC homeostasis [35,36]. Langerhans cells, a member of mononuclear phagocytic cells derived from bone marrow, are located in epithelium, mucosa, and immune organs [37]. There were a few MHCII^+^ Langerhans cells along with the limbal epithelial basal cells, in which they were defined as sentinel cells to process and present antigenic peptides, and mediate T-cell immune response [38,39].

### 2.2. Cell-Extracellular Matrix

Basement membrane (BM) is a component of extracellular matrix, which provides the structure for LEC anchorage. It is mainly composed of collagens and laminins, which exhibit heterogeneously in the cornea and limbus (Table 1) [40]. The limbal stroma has been reported to be highly innervated and vascularized [41], and to be closely related to the LSC phenotype [42,43]. Using a model of cross-culture of the corneal epithelium, limbal epithelium, corneal stroma, and limbal stroma, it was found that the limbal stroma induced the dedifferentiation of the corneal epithelium while corneal stroma promoted differentiation of limbal epithelium. Thus, limbal stroma and corneal stroma may possess the dedifferentiation and prodifferentiation potential, respectively [43]. Under the BM, a number of mesenchymal cells are located in limbal stroma. These cells penetrate the BM to make contact with LSCs by forming the calcium-dependent N-cadherin adhesion [44] and to reduce the differentiation of LSC by expressing CXCR4/SDF-1, maintaining the homeostasis of LSC [45]. They also suppress the T-cell immune response by inhibiting the proliferation of T lymphocytes [46], which plays an important role in corneal transplantation.

### 2.3. Cytokines

It has been reported that growth factors were involved in the processes of proliferation, differentiation, and self-renewal of LSCs [50]. Coexpression of nerve growth factor (NGF) with NGF receptors (NGFR) Trk A and p75 in the basal layer of LECs means that NGF may function as a critical autocrine or paracrine factor supporting stem cell self-renewal in the LSC niche [51]. Li et al. reported that transforming growth factor-α (TGF-α)/epidermal growth factor receptor (EGFR), IL-1β/IL1-R, and basic fibroblast growth factor (bFGF)/fibroblast growth factor receptor-1 (FGFR-1) were detected as more expressed in CECs compared to LECs. Among these cytokines and their receptors, the EGFR and bFGF showed specific expression in corneal epithelium [52]. TGF-α and IL-1β can serve as signals reflecting the epithelial healthy state and the epithelial being injured or under stress state, respectively. The greater expression of EGFR and IL1-R by CECs than LECs is attributed to modulating the differentiation of corneal epithelium. Similar to the expression pattern of EGFR and IL-1R, bFGF/FGFR-1 also mediates corneal epithelial differentiation by an autocrine method [52]. When the corneal epithelium is injured, insulin-like growth factor-I (IGF-I) will be rapidly overexpressed, and EGF and fibroblast growth factor-β (FGF-β) will also be produced. These increased cytokines act in different cellular processes for wound healing. In this system, the overexpressed IGF-I serves as stimulators to induce the expression of IGF receptor (IGFR) in limbal cells, leading to the differentiation of LSCs. Different to the IGF-I, the increased EGF and FGF-β are the main players in promoting the proliferation of limbal cells [53].

Imanishi et al. demonstrated the mechanism of EGF, keratinocyte growth factor (KGF), TGF-β, and platelet-derived growth factor (PDGF) in corneal wound healing [50]. Using different concentrations of these growth factors in a wounded cornea model, different effects of wound healing were shown. They found that natural PDGF and PDGF-BB enhanced the migration of endothelial cells only at the concentration of 3–10 ng/mL, whereas the cell migration could be suppressed when its concentration was increased. EGF promotes the growth of corneal keratocytes, corneal endothelial cells, and CECs with a dose-dependent manner within a particular concentration range, whereas over concentration will result in a decreased effect. Furthermore, Honma et al. demonstrated that TGF-β1 and TGF-β2 inhibited the proliferation effect of CECs enhanced by EGF, KGF and HGF [54]. These results suggest that the spectra of the involved cytokines with different concentrations at the different time points after wounding would have different impacts on the process of wound healing.

## 3. Dynamics of LSCs

**Figure 2:**
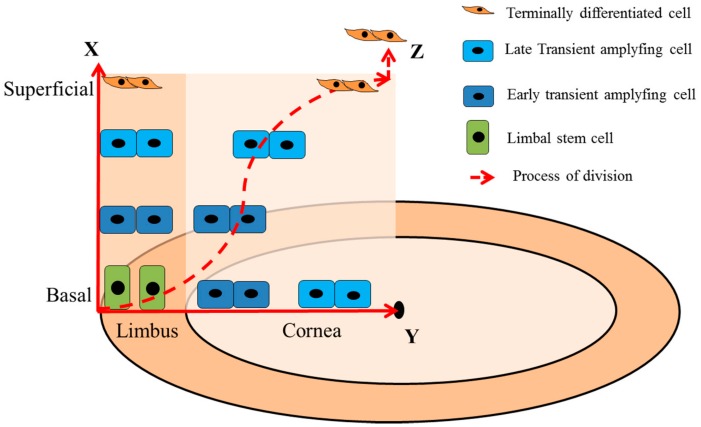
Simplified model of X/Y/Z hypothesis. X: anterior migration of the cells from the basal epithelium; Y: centripetal migration from the limbus; Z: desquamation from the surface. X + Y = Z.

LSCs play an important role in homeostasis of corneal epithelium. They divide into TACs by mitosis, retaining less stemness. Due to progressive loss of stemness along with multiple rounds of division, TACs finally divided into the terminally differentiated cells (TDCs) [30]. This process takes over two weeks [47]. The X/Y/Z hypothesis, proposed by Thoft et al., on the progress of LSC division is well recognized (Figure 2) [55]. They regard anterior migration from cells of the basal epithelium as “X”, centripetal migration from the limbus as “Y”, and desquamation from the surface as “Z”. The X/Y/Z hypothesis is related to the corneal epithelium being maintained, and cell loss must be balanced by cell replacement, i.e., X + Y = Z. The hypothesis has been proved by Kasetti et al. [7]. They found that the LSCs are located at limbus and progressively differentiate from limbus to the central cornea by using the transgenic mouse system. In the process of cornea wound healing, they found that LSCs were centripetal migration from the limbus and formed 2–3 layers of cells in the wound area at the healing beginning, and then gradually increased cell layers over time, eventually restoring the function of the cornea. Interestingly, they also found a fixed division capacity of TAC progenitors derived from LSC, which are subjected to the process of wound healing [7].

**Figure 3:**
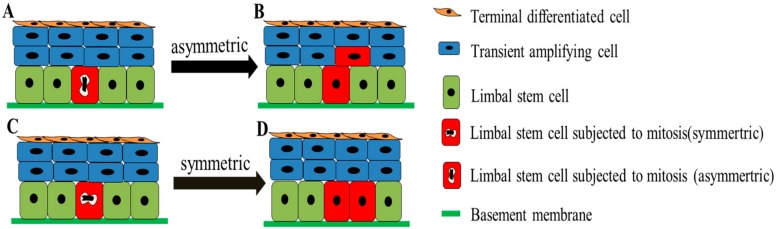
Simplified model of symmetric and asymmetric division of LSCs. When the LSC (red) undergoes mitosis, it can be subjected to asymmetric division (**A**) to divide into a stem cell remaining anchored on the BM and a daughter cell detaches from the BM and migrates forward to be a TAC (**B**), or to asymmetric division (**C**) to divide into two similar daughter stem cells both remaining anchored on the BM (**D**).

Furthermore, Lamprecht proposed that there are two different forms of basal cell division in the corneal epithelial population: (1) Symmetry division: The corneal epithelial basal cells divide into two morphologically similar daughter cells, eventually anchoring to the BM; (2) Asymmetric division: the corneal epithelial basal cells divide into two morphologically dissimilar daughter cells, one of which remains anchored on the BM and the other detaches from the BM and migrates to the suprabasal layer of corneal epithelium becoming a TAC (Figure 3) [56]. Additionally, Lobo et al. reported that the death of LSCs can stimulate the division of LSCs in neighbors. In most cases, the dead LSC would be replaced by the one daughter cell of a neighboring LSC undergoing symmetry division. However, 5–10% of the time, both neighbors respond synchronously, producing two LSCs. One replaces the dead cell, while the excess LSCs are pushed into the cornea [57].

## 4. Putative Biomarkers of LSC

Many putative LSC biomarkers have been reported (Table 2) including cell cycle regulators, cytoskeletal proteins, cell adhesion molecules, enzymes, growth factors and their receptors, ATP-binding cassette transporters and differentiation associated markers [58,59,60].

### 4.1. Cell Cycle Regulators

#### 4.1.1. ΔNp63α

As a member of the p53 family, p63 plays an important role in cell cycle regulation such as cell proliferation and apoptosis. The *p63* gene encodes two groups of protein isoforms, namely TAp63 and ΔNp63. These two groups are distinguished by the structure of the N-terminal domain. TAp63 group contains a complete transactivation-competent (TA) dominant with homology to p53, which exhibits tumor suppressor properties. ΔNp63 group contains a truncated dominant ΔN at its N-terminus, which exhibits oncogenic activities [71]. Alternative splicing at the C termini of both groups generates three different isoforms, α, β, and γ, in each of TAp63 and ΔNp63 [72]. Pellegrini et al. reported that p63 was expressed in the basal layer of LECs but not in the corneal epithelium [73]. ΔNp63α has been reported to be able to induce cell cycle arrest and apoptosis and differentially regulate endogenous p53 target genes [74]. Expression of ΔNp63α was specifically detected in the limbal basal cells, which indicated that ΔNp63α may be a putative biomarker of LSC [75].

#### 4.1.2. C/EBPδ

The CCAAT/enhancer-binding protein (C/EBP) members belong to a family of basic region leucine zipper transcription factors. C/EBPδ is one of the six members of the C/EBP family, expressed in various tissues and cell types, and involved in the cellular processes such as proliferation, differentiation, metabolism, and inflammation [76,77]. It regulates the cell cycle by inducing G0/G1 arrest, especially in the epithelial cells. As reported, p27Kip1 and p57Kip2 were highly expressed in the nucleus when the cells were subjected to mitotic arrest, highly expressed in the cytoplasm at G1/S, and not expressed when the cells were subjected to the proliferation [78]. When C/EBPδ is expressed, LSCs activate the expression of p27Kip1 and p57Kip2 to prolong its cell cycle without the proliferative capacity changing [79]. Moreover, expression of p27Kip1 and p57Kip2 were detected in the nucleus in C/EBPδ^+^/ΔNp63α^+^ cells, and in the cytoplasm in C/EBPδ^−^/ΔNp63α^+^ cells by using the immunofluorescence technique. These indicated that C/EBPδ is a candidate biomarker of G0 LSCs [79].

### 4.2. ATP-Binding Cassette Transporters

#### 4.2.1. ABCG2

ABCG2, a member of the ATP-binding cassette transporter family, serves as a specific biomarker for bone marrow stem cells. Goodell successfully isolated the mouse bone marrow stem cells based on the efflux of Hoechst 33342, the DNA-binding dye, by ABCG2 [80]. De Paiva et al. found that ABCG2 was specifically expressed in limbal basal cells, and about 2.5–3% ABCG2^+^ cells there were isolated by fluorescence activated cell sorting (FACS) [81], which is consistent with the expected numbers of LSCs. Thus, ABCG2 was presumed as a biomarker of LSC [28,82].

#### 4.2.2. ABCB5

As another member of the ATP-binding cassette transporter family, ABCB5 has been reported frequently in the investigations of cancer target therapy. Wilson et al. reported that ABCB5 was significantly upregulated in colon and rectal cancer cells and ABCB5^+^ tumor cells showed apoptosis resistance [83], suggesting that ABCB5 can be a therapeutic target against colon and rectal cancer. Recently, *Abcb5* has been reported to be a necessary gene for LSC development and repair [8]. Similar to *Abcb5* being coexpressed with bromodeoxyuridine (BrdU) label-retaining LSCs in mice, it was also found to be coexpressed with p63α^+^ LSCs in humans. Both ABCB5^+^ cells in mice and humans were located in basal limbal epithelium, which means that ABCB5 may be a putative biomarker for LSC. Furthermore, lower populations of ABCB5^+^ LSCs in LSCD patients were found compared to healthy people. These ABCB5^+^ LSCs have been proved to have an ability to recover vision in autologous and allogeneic corneal transplantation mouse models. It has been reported that knockout of *Abcb5* genes in mice induced a decreasing trend of LSC, which accounted for the depletion of quiescent LSCs due to enhanced proliferation and pro-apoptotic *p53* and downregulating antiapoptotic *Bcl2* and *Bcl-x*, leading to defective corneal differentiation and wound healing [8].

### 4.3. Cytoskeletal Proteins

#### 4.3.1. CK5 and CK14

Cytokeratin (CK) 14, a member of the type I keratin family, is an intermediate filament protein mainly expressed in the cells of the basal layer of the stratified epidermis. It serves as an LSC putative biomarker [84]. Usually, CK14 forms a dimer with CK5, a type II keratin, to be the main component of epithelial cytoskeleton. Zhao et al. demonstrated that CK5/CK14 was specifically expressed in the limbal epithelial basal cells, whereas CK3/CK12 was expressed all over the corneal epithelium and in the limbal superficial cells. This finding indicated that CK5^+^/CK14^+^ and CK3^−^/CK12^−^ can be used to accurately identify the LSCs [85]. Moreover, CK14 has been described to be associated with proliferation of epithelial cells and related to the ectoderm development of the fetal epidermis [86]. Considering that the corneal epithelium and the epidermis are both developed from the common embryonic ectoderm, CK14 may play an important role in the development of corneal epithelium [86]. Furthermore, Meller et al. demonstrated that CK14 was involved in the formation of hemidesmosomes, a key structure for basal cells to anchor to the BM [87].

Chen et al. reported that CK5/CK14 were restricted to the basal layer cells of a quiescent bovine limbal epithelium [88], which is consistent with the reported that CK5/CK14 can be thought of as an LSC putative biomarker [85]. However, there are limits of CK5/CK14 to be LSC biomarkers. They also found that, when LSCs undergo the artificially promoted cell differentiation by air-lifting after two weeks cultured on amniotic membrane (AM), the expression of CK5 and CK14 showed no decrease and actually slightly increased, due to the dynamic status of the LSC differentiation states [88]. It suggested that CK5/CK14 can be thought of as LSC biomarkers under specific circumstances, such as no differentiation force on LSC.

#### 4.3.2. Vimentin and Cytokeratin 19

CK19, the biomarker of bile duct cells, hepatic progenitor cells (HPCs), and early hepatocytes, is clinically related to the diagnosis and prognosis of hepatocellular carcinoma (HCC) [89]. It was also described as a biomarker of epithelial stem cells of skin and to be related to the proliferation of cells. As reported, CK19 is expressed in the limbal epithelial basal cells as a putative biomarker of LSCs [90,91]. However, its specificity is controversial. Chen et al. found that CK19 was not only expressed in LECs but also in CECs [27]. Similarly, Ramirez-Miranda et al. detected the expression of CK19 both in limbal and conjunctival epithelial cells [92].

The cytoskeleton, responsible for the stability of the cell structure, contains microfilament, microtubules and intermediate filaments. Vimentin is the most abundant protein in the intermediate filament [93,94]. It has been reported to be associated with the adhesion, migration and invasion, signaling transmission, differentiation of cells, and cytoskeletal rearrangement, and the regulation of cell morphology [95,96,97,98]. The expression of vimentin was detected in limbal epithelial basal cells [59], whereas it usually combines with the expression of other putative biomarkers such as P63 and ABCG2, etc., for the identification of LSCs [48,99].

### 4.4. Differentiation Associated Proteins

#### 4.4.1. Cx 43

Connexin (Cx) 43, known as GJA1 (gap junction alpha-1), is a member of the gap junction protein family, which was encoded by *Gja1* gene located human 6th chromosome. Cx consists of two extracellular loops, four membrane-spanning domains, one cytoplasmic loop, one N-terminal tail, and one C-terminal tail. Six connexins make up a connexon. Two neighboring corneal epithelial cells can be connected between their cytoplasms by a connexin channel, which consists of two connexons or hemichannels from the two adjacent cells, respectively. Gap junction is assembled with hundreds of connexin channels to regulate the transport of ions, metabolites, and low molecules, thereby regulating cells’ proliferation, differentiation, and regeneration [100,101]. Chen et al. reported that the expression of Cx43 was detected in both the central CECs and the suprabasal cells of limbal epithelium but not in limbal epithelial basal cells by immnofluorescence staining [102]. Moreover, they found that these Cx43^-^ cells showed p63^+^/ABCG2^+^/Integrin β1^+^/CK3^+^/Involucrin^+^ phenotype [102], indicating that Cx43 may serve as a putative negative biomarker of LSC.

#### 4.4.2. CK3 and CK12

CK3 and CK12, the intermediate filament proteins, are encoded by the highly conserved genes of *Ck3* and *Ck12*, respectively, are the main components of cytoskeleton functioning to stabilize the structure of CECs. CK3 and CK12, serving as differentiation biomarkers of CEC, are widely recognized to be specifically expressed in CECs, limbal suprabasal cells and not in the limbal basal cells, which are thought of as LSCs [59]. Moreover, CK12 and CK14 were used as the biomarkers of corneal differentiated cells and corneal progenitor cells, respectively, to determine the kinetics of corneal epithelium differentiation [103]. The amount of CK12^+^ and CK14^+^ cells from the mice CECs’ suspension were counted by double-immunofluorescence staining at various postnatal age groups. The results showed that 70% cells exhibited CK12^+^/CK14^+^ phenotype and 30% exhibited CK14^+^ until six months after birth, which indicated that a small population of corneal basal cells would maintain the stem cell characteristic until six months after birth [103].

## 5. Identification of LSCs

LSC plays an important role in the regeneration of damaged corneas. The LSC-based transplantation, the expanded culture of LSCs from patients in vitro for autologous transplantation, has been applied in the clinic. The investigation by Rama et al. showed that more or less 3% of p63^+^ cells out of the total number of candidate cells for corneal transplantation were related to a successful rate of 78% or 11% of patients, respectively [17]. These data suggested that the ratio of LSCs to total candidate transplantation cells was significantly associated with the effective of corneal transplantation [17]. Hence, the accuracy of identified LSCs is urgent for corneal transplantation. However, there is still no LSC-specific biomarker for accurate identification yet.

As reported, identification of LSC is usually based on the following methods:

1. The coexpression of LSC biomarkers: such as p63^+^, ABCG2^+^, integrin α9^+^, vimentin^+^, Cx43^−^, CK3^−^/CK12^−^, and involucrin^−^, etc.;
2. N/C ≥ 0.7: Schlotzer-Schrehardt et al. demonstrated that the ration of N/C of LSC on the BM was higher than TACs and CECs, respectively [59]. Priya et al. successfully identified and quantified LSCs based on ABCG2 and N/C ≥ 0.7 [69]. Additionally, Kasinathan et al. established a two-step protocol by combining basal cell isolation and laser capture microdissection (LCM) of small cells with N/C ≥ 0.7 for LSC enrichment, which achieved 76–78% enrichment of LSC from 2% LSCs in total LECs [104];
3. Label-retaining cell: bromodeoxyuridine (BrdU) can take the place of thymidine to be incorporated into the replicated DNA during the S-phase of the cell cycle. The BrdU-based “pulse-chase” experiment has been widely applied for the identification of stem cell. After a period of BrdU pulse, all cells with different degrees of differentiation can be labeled with BrdU. Slow cell cycling is a characteristic of LSCs, which means less division of BrdU labeled LSCs compared to the differentiated cells in the same time. Thus, after a period of BrdU chase, the BrdU-retaining cells can be considered as LSCs [105];
4. Side population (SP) phenotype: SP is a sub-population of Hoechst blue^−^/Hoechst red^−^ cells outside the main population based on staining with DNA-binding dye Hoechst 33342 and isolating by FACS. SP phenotype has become a characteristic of stem cells. Goodell et al. successfully isolated mice bone marrow stem cell by using FACS based on Hoechst-SP method, the Hoechst 33342 efflux activity resulting from the specific protein expression of stem cells, such as ATP binding cassette transporters [80]. Similarly, LSCs showed the ABCG2^+^/ABCB5^+^ phenotype. By this method, Shaharuddin et al. successfully isolated the LSCs [106].

## 6. Difficulties in the Study of LSC-Specific Biomarkers

### 6.1. Particularity of Limbal Histochemistry

Horizontally, limbus showed a narrow zone and unclear boundaries. Histologically, there are some differences between the cornea and limbus, such as the specific “Vogt” niche in limbal epithelium and more layers of LECs than cornea [58,107]. However, it is difficult to distinguish limbal epithelium from corneal epithelium. Chen et al. reported that CK12 specifically expressed in LECs but not in conjunctiva [108]. Latta et al. demonstrated that CK3 and CK12 as differentiation-related molecules were specifically expressed in CECs but not in LECs [18]. However, Ramirez-Miranda et al. reported that CK12 was not only expressed in cornea CECs but also in LECs [92]. It seems a good approach to distinguish the limbus from the conjunctiva based on goblet cells, which were used to diagnose LSCD [109]. Controversially, the Stevens–Johnsons syndrome and long-term administration of glaucoma drugs could lead to LSCD with no goblet cells invasion [110]. Some investigators reported that CK19, CK13 and MUC5AC can serve as biomarkers of conjunctival epithelial cells [110,111], but others argued that the expression of CK19 was detected both on the limbal and CECs [27]. Although Ramirez-Miranda et al. believed that CK13 was more specific than CK19 in terms of identification of conjunctival epithelial cells [92], Poli et al. showed that CK13 was expressed in both the conjunctival epithelium and the suprabasal and superficial layers of the limbal epithelium [110].

### 6.2. Influences of Enzyme Digestion

The limbal epithelial suspension, used for the isolation of LSCs, is usually prepared from limbus mass based on the digestion of enzymes, such as dispase II, collagenase A and trypsin [112,113]. Dispase II destroys the BM, which induces the separation of limbal epithelium from the stroma. Collagenase A is able to digest the extracellular matrix to disrupt the cellular connection in a limbal niche. Trypsin plays a role in the separation of the cells mass into a suspension of single cells [113,114]. There were many reports demonstrating the optimization of digest conditions, such as digestion time and the concentration of enzymes to avoid enzymes destroying the integrity of LSCs [115,116]. Incubation of LSCs in enzyme digestion solutions for a long time may cause the destruction of extracellular peptides. On the other hand, dispersing the limbal epithelium into single cell suspension will destroy the LSC niche such as the connection of cell–cell and cell–matrix, which plays an important role in the maintenance of stemness [117].

### 6.3. Lack of Robust LSC Isolation Technology

Complicated structures are organized around the limbus including the cornea, sclera, and conjunctiva [118,119]. A small amount of LSCs are hidden among the LECs, which account for only 0.5–10% [18], resulting in the difficulties with isolating the LSCs with such low frequency from such complex limbal epithelium suspension. As reported, there were some traditional methods to isolate the LSCs such as gradient centrifugation [120], FACS [121], and magnetic-activated cell sorting (MACS) [64]. All of these methods are based on enzyme digestion, which potentially changes gene expression patterns and protein profiles of LSCs, and increases the risk of contamination of suspension due to complicated operations [122,123,124].

The laser capture microdissection (LCM) technique, first proposed by Emmert-Buck et al., has been employed to isolate target cells from complex and heterogeneous tissues. The operation procedures of this technique require preparing a tissue section from the target tissue, in order to apply a transparent thermoplastic film to the tissue section, then to locate the target cell under a microscope and make a mark with the aid of a computer [123]. The film covered by the labeled cells is heated by a laser pulse. The selected cells are adsorbed by a strong adhesion force. The collected cells can be used for downstream experiments. The combination of LCM with the next-generation sequencing (NGS) has become a hot topic of research into heterogeneity and specific biomarkers. Bath et al. collected four distinct human limbal compartments, the basal limbal crypts, the superficial limbal crypts, the paracentral/central corneal epithelium, and the adjacent limbal stroma, for NGS by LCM [125]. The abundant transcripts from these four discrete compartments provided plentiful information for the study of LSC biomarkers. Polisetti et al. demonstrated the connection between cell adhesion molecules and LSC niches by LCM. They collected cells from 500 human limbal and corneal frozen sections, respectively, for RNA extraction following qPCR and immunofluorescence detection [64]. With this technique, they successfully demonstrated the relationship between LSC niche cells (LSCs, melanocytes, mesenchymal cells, immune cells) and the adhesion profile of basal cell-BM.

However, there are still some limits of LCM. It needs tissue samples to be made into formalin-fixed paraffin-embedded (FFPE) or optimal cutting temperature (OCT) compound-embedded frozen sections, usually 5–15 μm [126]. As reported, the average sizes of limbal basal epithelial cells and corneal basal epithelial cells were 10.1 ± 0.8 μm and 17.1 ± 0.8 μm, respectively [28]. Therefore, preparation of such thin sections may cut the corneal basal epithelial cell into several parts, resulting in incomplete RNA information that can not represent a full corneal basal epithelial cell. Moreover, since little RNA was obtained each time from the slice of LCM, a large number of sections need to be prepared for obtaining enough RNA for future studies. Additionally, pre-treatment of sections for LCM, such as fixative, staining, and dehydration, can significantly influence the RNA quality [127], reducing the efficiency of subsequent experiments, such as NGS and microarray analysis [128]. Many dyes were used for staining during LCM; however, some of these have negative effects, such as eosin having the ability to interfere with two-dimensional gel electrophoresis (2-DE) [129]. The disadvantages of FFPE tissue for LCM are cross-links of nucleic acids–proteins and proteins–proteins [130], leading to proteins not being able to be extracted completely. In spite of frozen tissue being excellent for nucleic acids and protein isolation, a lack of histologic differentiation and frequently having incomplete tissue dehydration result in no adherence of the cells to the membrane, leading to a failure of removing target cells from slides [126].

### 6.4. Heterogeneity of LSCs

Heterogeneity has been reported in various types of stem cells, such as embryonic stem cells and mesenchymal stem cells [131,132]. LSCs also exhibit heterogeneity [133]. Lin et al. observed the morphology and measured the epithelial thickness of human limbal palisades of Vogt (POVs) by using the spectral-domain optical coherence tomography (SD-OCT) [134]. They found that the appearance of POVs and the thickness of limbal epithelium showed a big difference in different age groups and anatomical regions, suggesting that age and anatomic regions had significant effects on the microstructure of limbus. Hence, it was proposed that the heterogeneity of LSCs was mainly attributed to age groups and anatomical regions.

Aging showed not merely a morphological influence on LSCs, but also on the expression pattern of the related genes. Notara et al. reported that the phenotype of LSC changes with aging. Humans who are over 60 years of age showed drastically reduced crypts in the limbus, which were closely related to the LSC niche [135]. Tanifuji-Terai et al. reported the kinetics of corneal epithelium differentiation by monitoring the expression of CK12 and CK14 in mice corneal epithelium from embryo to postnatal 180 days [103]. The results demonstrated that the expression of CK12 and CK14 showed dynamics with age. Many reports about the distribution of crypts in superior, inferior, nasal, and temporal regions of limbal epithelium were consistent with the view that the number of crypts in the superior and inferior regions are more than that in nasal and temporal [136,137,138]. Zhao et al. accurately counted LSCs in superior, inferior, nasal, and temporal of limbal epithelium based on the characteristic that BrdU labeled of LSCs [139]. Their results suggested that LSCs in the superior region were more than that in the inferior, and the temporal LSCs were more than the nasal. Moreover, among the four regions, the superior temporal region showed the greatest abundance of LSCs, and the inferior nasal region displayed the least LSCs.

All in all, aging and anatomical regions are closely associated with the numbers, distribution, expression profile, and crypt of LSCs. In the recent reports, the most samples of cornea, limbus, and conjunctiva are widely derived from donation and/or patient’s pathological tissue mass. Such sources of samples showed the randomness of age and anatomical region, which is against the heterogeneity of LSCs. Moreover, the freshness of samples may also be related to the expression profile of LSCs.

## 7. Prospectives

### 7.1. Development of mRNA- or microRNA-Based Biomarkers

In the past few decades, the research on LSC biomarkers based on proteins has been widely reported; however, the LSC-specific biomarker still has not been reported. Currently, the NGS technique has been widely developed and applied in biomedicine research. It suggested that there are more opportunities to obtain the mRNA and microRNA information. Recently, many reports have demonstrated the new biomarkers at the RNA level. Howlett et al. reported that microRNA 8059 can be considered as a biomarker for the coronary artery calcification [140]. Shen et al. suggested that hsa-miR-320d and hsa-miR-582 can be considered as biomarkers of aortic dissection regulating apoptosis of vascular smooth muscle cells [141]. Li et al. reported that the MicroRNA-192-5p served as a biomarker of survival for Stage IIIB colon cancer patients [142]. The development of new biomarkers in medicine based on mRNA and microRNA provide a new direction for LSC-specific biomarker study.

### 7.2. Application of In Situ Sequencing Technology

For the heterogeneity of LSCs in anatomical regions, it is critical to ensure spatial information for samples. The RNA sequencing technology is based on the purified nucleic acids, indicating the mixing of specific regional information. The in situ hybridization has been applied to the localization of LSCs [27]. However, it is limited to relying on known sequences. In 2013, Ke et al. first developed a new technology, known as in situ sequencing, for RNA analysis [143]. They designed a padlock probe to hybridize its both ends with the target sequence to form a loop template for replication by rolling circle amplification (RCA) technology. By this method, we are able to gain the target RNA sequence with the information of original morphology and location of its hosting cells [143]. Hence, the application of in situ sequencing technology may solve the location and heterogeneity problems of LSCs.

### 7.3. Application of the Inducible Transgenic Animal System

The microenvironment of the LSC niche is the key for maintaining LSC stemness. The method of isolation of LSCs may destroy their microenvironment. The application of BrdU pulse-chase to identify LSCs has demonstrated the importance of animal models well [8,139]. Recently, Sartaj et al. developed a tetracycline-inducible (tet-off) double transgenic “pulse-chase” mouse system (K5Tta × TRE-H2BGFP) that accurately identified LSCs, and then isolated the LSCs, combined with NGS technology, to analyze the expression profile of candidate biomarkers of LSC [144]. Such a similar transgenic mouse system has also been reported to demonstrate the mechanism of LSC-promoting wound healing [7,145] and the homeostasis of corneal epithelium [84]. Therefore, study on LSC in the transgenic animal system may be an excellent method to avoid the destruction of LSC niche microenvironments.

### 7.4. Development of Virtual Simulation Technology

The application of interdisciplinary technology has been rapidly developed. There are many reports on application of virtual simulation technology to solve medical problems [146,147]. Recently, virtual simulation technology also has been applied for the study of LSCs. Molvær et al. simulated the 3D computer model of the human corneolimbal region by 3D visualization software based on series hematoxylin and eosin (HE) stained paraffin sections [148]. Using this technique, they successfully identified three niche types of LSCs (limbal epithelial crypts, limbal crypts and focal stromal projections), and their distribution in superior, inferior, nasal, and temporal regions. Lobo et al. successfully demonstrated the mechanism of self-organization centripetal migration of LSCs to repair the wounded corneal epithelium without external cues by using the virtual digital model [57]. In a word, combining traditional biotechnologies with the virtual simulation technology to study LSCs showed great advantages. This reduces the amount of animals used in experiments. Moreover, the computer provides advantages to execute multiple complex bio-hypotheses at the same time.

## 8. Conclusions

LSC-based transplantation has been reported for treatment of bilateral LSCD. The clinical results showed that the ratio of LSCs to total number of candidate transplantation cells has a significant influence on the effect of LSC-based transplantation, which suggests that it is important to accurately identify and isolate the LSCs. However, there are still no reliable LSC-specific biomarkers. Here, we summarize the current views to provide an insight into LSC putative biomarkers, such as ΔNp63α, C/EBPδ, ABCG2, ABCB5, CK5, CK14, Cx43, CK3, and CK12, etc., which were associated with the cell cycle, proliferation, and differentiation of LSCs. We also summarize the methods of identification of LSCs based on stem cell characteristics, co-expression of putative biomarkers, N/C ≥ 0.7, BrdU label retaining, and SP phenotype. Additionally, the common obstacles of LSC-specific biomarker research were analyzed. These were attributed to the particularity of limbus anatomy and histochemistry, being insufficient overall, heterogeneity of LSCs, lack of robust LSCs isolation techniques, the influence of enzyme digestion and the destruction of the LSC niche, and low RNA quality for future experiments such as NGS and microarray analysis. We recommend approaches to explore mRNA- or microRNA-based biomarkers of LSC, application of in situ RNA sequencing technology, which preserves the target cells’ regional information, and transgenic animal system to ensure no destruction of LSC niches, and a combination of traditional technologies with virtual simulation technology to shed more light on LSC biomarkers.

## Figures and Tables

**Table 1:** Distribution of collagens and laminins on cornea and limbus.

BM Components	Cornea	Limbus	References
α1(IV) collagen chain	−	++	[47]
α2(IV) collagen chain	−	++	[47]
α3(IV) collagen chain	−	−	[47]
α4(IV) collagen chain	−	+	[47]
α5(IV) collagen chain	++	++	[47]
α6(IV) collagen chain	++	++	[48]
Type V collagen	+	−	[48]
Type VI collagen	−	−	[48]
Type VII collagen	++	++	[48]
Laminin α1chain	+	+	[49]
Laminin α2 chain	±	+	[49]
Laminin α3 chain	++	++	[49]
Laminin α4 chain	−	±	[49]
Laminin α5 chain	−	+	[49]
Laminin β1 chain	++	++	[49]
Laminin β2 chain	+	++	[49]
Laminin β3 chain	+	++	[49]
Laminin γ1 chain	+	++	[49]
Laminin γ2 chain	+	++	[49]
Laminin γ3 chain	±	+	[49]

Note: −, no expression; ±, weak expression; +, moderate expression; ++, strong expression.

**Table 2:** The expression of putative biomarkers in cornea and limbus.

Group of Putative Biomarkers	Putative Biomarkers	Corneal		Limbal		References
		Basal	Suprabasal	Basal	Suprabasal	
Cell structural proteins	Vimentin	−	−	++	+	[59]
	CK5/14	−	−	+	+	[61]
	CK19	+++	+++	+++	+	[62]
	CK15	+	−	++	−	[63]
Cell adhesion molecules	Integrinα2	+++	+++	+++	+++	[64]
	Integrinα3	+++	+	+++	±	[64]
	Integrinα4	−	−	+	±	[64]
	Integrinα6	++	+	−	+++	[64]
	Integrinα8	−	−	±	±	[64]
	Integrinα9	−	−	+++	±	[65]
	Integrinβ1	+++	++	+++	+	[27]
	Integrinβ4	++	+	−	+	[59]
	P−cadherin	±	−	±	−	[59]
	E−cadherin	+	+++	−	+++	[36]
	N−cadherin	−	−	+	±	[36]
	Frizzled7	+	−	+++	++	[66]
Enzymes	α−enolase	++	+	+++	+	[65]
	cytochrome oxidase	++	+	+++	+	[67]
	Na^+^/K^+^−ATPase	++	+	+++	+	[67]
Growth factors and its receptors	EGF−R	+++	+++	+++	+	[27]
	KGF−R bek	±	−	−	−	[59]
	NGF−R TrkA	±	−	+	−	[51]
	NGF−R p75	++	−	++	−	[51]
	NGF	+	±	++	−	[51]
Cell cycle regulators	ΔNp63α	−	−	+++	±	[68]
ATP−binding cassette transporters	ABCG2	−	−	+++	±	[69]
	ABCB5	−	−	+++	++	[8]
Differentiation associated proteins	Cx43	+	+++	−	+++	[69]
	CK3/12	+++	+++	−	+++	[59]
	Involucrin	+	+++	−	+++	[70]

Note: −, no expression; ±, very weak expression; +, weak expression; ++, moderate expression; +++, strong expression.

## Abbreviation

The following abbreviations are used in this manuscript:

2-DE	Two-dimensional gel electrophoresis
AM	Amniotic membrane
bFGF	Basic fibroblast growth factor
BM	Basement membrane
BrdU:	Bromodeoxyuridine
C/EBP	CCAAT/enhancer-binding protein
CECs	Corneal epithelial cells
CK	Cytokeratin
Cx	Connexin
EGFR	Epidermal growth factor receptor
ESCs	embryonic stem cells
FACS	Fluorescence activated cell sorting
FFPE	Formalin-fixed paraffin-embedded
FGFR-1	Fibroblast growth factor receptor-1
FGF-β	Fibroblast growth factor-β
GJA1	Gap junction alpha-1
HCC	Hepatocellular carcinoma
HE	Hematoxylin and eosin
HPCs	Hepatic progenitor cells
iPSCs	Induced pluripotent stem cells
IGF-I	Insulin-like growth factor-I
IGFR	IGF receptor
KGF	Keratinocyte growth factor
LCM	Laser capture microdissection
LECs	Limbal epithelial cells
LSCs	Limbal stem cells
LSCD	Limbal stem cell deficiency
MACS	Magnetic-activated cell sorting
NGF	Nerve growth factor
NGFR	NGF receptors
NGS	Next-generation sequencing
N/C	Nuclear-to-cytoplasm
OCT	Optimal cutting temperature
PDGF	Platelet-derived growth factor
PSCs	Pluripotent stem cells
POVs	Palisades of Vogt
RCA	Rolling circle amplification
SD-OCT	Spectral-domain optical coherence tomography
SP	Side population
TACs	Transient amplifying cells
SESCs	Skin epithelial stem cells
TDCs	Terminally differentiated cells
TGF-α	Transforming growth factor-α
WHO	World Health Organization.
